# The VALUE Study: Exploring the Value of a Clinical Ethics Consultation Service at the “A. Gemelli” Hospital

**DOI:** 10.3390/healthcare14030395

**Published:** 2026-02-04

**Authors:** Salvatore Simone Masilla, Barbara Corsano, Simona Giardina, Costanza Raimondi, Pietro Refolo, Dario Sacchini, Clara Todini, Antonio G. Spagnolo

**Affiliations:** 1 Research Centre for Clinical Bioethics & Medical Humanities, Università Cattolica del Sacro Cuore, Largo Francesco Vito 1, 00168 Rome, Italypietro.refolo@unicatt.it (P.R.); clara.todini@unicatt.it (C.T.); antoniogioacchino.spagnolo@unicatt.it (A.G.S.); 2 Department of Health Care Surveillance and Bioethics, Section of Bioethics and Medical Humanities, Università Cattolica del Sacro Cuore, Largo Francesco Vito 1, 00168 Rome, Italy; 3 Fondazione Policlinico Universitario Agostino Gemelli, Largo Francesco Vito 1, 00168 Rome, Italy; barbara.corsano@unicatt.it

**Keywords:** clinical ethics consultation, service evaluation, qualitative study, therapeutic proportionality, shared care planning, medico-legal issues

## Abstract

**Background:** Clinical Ethics Consultation (CEC) helps healthcare professionals, patients, and families address ethically complex situations in clinical practice. Since 2016, requests for CEC at the Fondazione Policlinico Universitario “A. Gemelli” IRCCS (FPG) have been entered into the hospital IT system like other consulting services. This has increased both the number of requests and the need to monitor and evaluate the service. **Aims:** This qualitative study investigates how the CEC service at FPG is perceived in terms of its value, role, and impact, and further aims to identify appropriate strategies for evaluating the service. **Methods:** Semi-structured interviews were conducted with 28 healthcare professionals who had used or taken part in the service within the previous two years. Data were examined using reflective thematic analysis, leading to the development of codes and themes. **Results:** Five main themes emerged: (1) Role and identity of the clinical ethics consultant the, (2) Benefits of CEC for clinical practice, (3) How to evaluate the CEC Service, (4) HCPs’ Evaluation, and (5) CEC Service Improvement Strategies. Participants saw the consultant as an “active third party” who combines ethical, clinical, and communication skills to mediate conflicts, support teamwork, and guide shared care planning. The service was viewed as strengthening patient-centered care by improving communication with patients and families, clarifying treatment proportionality, and reducing clinicians’ decisional isolation. Interviewees emphasized the need for structured evaluation tools that include both quantitative indicators and qualitative feedback. Satisfaction with the service was consistently high, with recommendations to expand consultant availability, improve timeliness, and enhance training. **Conclusions:** CEC appears to serve as a clinical, relational, and training resource that fosters ethically grounded, collaborative, and person-centered care.

## 1. Introduction

According to the American Society for Bioethics and Humanities (ASBH), Clinical Ethics Consultation (CEC) can be defined as a service “carried out by an individual consultant, a group, or a committee to address the ethical issues involved in a specific clinical case. Its primary purpose is to contribute to patient care in both manner and outcomes, through the identification, analysis, and resolution of ethical issues” [1,2]. CEC is therefore a specialized service aimed at helping doctors, nurses, and other healthcare professionals, as well as patients, family members, and healthcare managers, to address moral conflicts that arise in clinical practice [3].

In the United States [4], and in other Western countries (such as the United Kingdom), dedicated CEC services have long existed and are now present in most hospitals. The Joint Commission on Accreditation of Healthcare Organizations, an independent, nonprofit organization that accredits and certifies hospitals and other healthcare institutions [5], requires each facility to identify a permanent contact responsible for addressing ethical issues within the organizational and clinical context. Despite international interest in the service, in Italy healthcare ethics consulting remains limited to a few hospitals [5,6].

At FPG, the CEC Service is provided by consultants from the Bioethics and Medical Humanities section of the Università Cattolica del Sacro Cuore in Rome, under an agreement between the university and the hospital. This arrangement ensures support in addressing ethically complex situations that may arise in clinical practice. The service has been operating continuously since 1992. Moreover, since 1 January 2016, it has been integrated into the hospital’s IT System allowing for the timely submission, reception, management and fulfillment of consultation requests from clinical departments [3].

At FPG, CEC requests are currently submitted through the hospital’s IT system, following the same procedure used for any other specialist consultation. As a result, physicians in each department are the ones who formally initiate the request through this official channel. In many cases, they act as intermediaries, conveying ethical or clinical questions raised by nurses, other staff members, patients, or family members. In addition, a telephone on-call service remains available, allowing other users to access the consultation when needed without requiring the direct involvement of a physician.

The Clinical Ethics Consultation (CEC) service evaluated in this study operates as a bedside-oriented consultation service integrated into routine clinical practice. Consultations are typically conducted by one or more members of a multidisciplinary team composed of physicians—primarily specializing in forensic medicine—and philosophers with formal training in bioethics. All consultants are affiliated with the Department of Security and Bioethics at the Catholic University of Rome, which provides a shared ethical framework and methodological orientation. The consultation model is primarily case based, grounded in Jonsen’s Four Boxes method, and emphasizes direct interaction with healthcare teams, patients, and families [4].

According to an interim analysis of a retrospective study recently approved by the ETCO Committee “Lazio Area 3” (protocol no. 0001504/25), the service handled approximately 160 consultations between 2024 and 2025.

Consultations may address ethical issues arising in clinical practice or support healthcare teams in the development of a shared treatment plan.

To date, reports conducted over time [3,7,8], show a steady increase in requests for CEC at FPG, as along with the involvement of an expanding number of healthcare professionals in shared decision-making processes supported by the presence of a bedside consultant.

Over the years, the need to evaluate the service itself has emerged, both to identify and address its weaknesses and to strengthen its positive aspects. However, this evaluation process is inherently complex, as the service relies on narrative and dialogical approaches [9,10]. Moreover, there is currently no unified or well-calibrated system for assessing the different CEC models [11]. This limitation reflects the heterogeneity of methodological approaches [12], and the diversity of ethical and cultural perspectives [5].

We considered it necessary to conduct an exploratory qualitative study involving physicians and healthcare professionals who request consultations or have previously taken part in decision-making processes supported by the service. The study was approved by the Lazio Area 3 Territorial Ethics Committee on 28 November 2024 (ref. No. 0001883/24).

## 2. Objectives

The primary objective of this study is to qualitatively assess the impact of the CEC service on healthcare professionals who have accessed it for decision-making support, as well as its overall influence on care processes.

The secondary objective is to explore potential strategies for evaluating and further improving the service.

## 3. Methods

To achieve the proposed objectives, a qualitative research strategy [13] was defined, consisting of semi-structured, face-to-face interviews with physicians and healthcare personnel involved in the clinical ethics consultancy service. The interviews were conducted by two researchers (SSM and CT) who were also engaged in participatory observation of the service. Both conducted their research as part of their doctoral studies. CT has a background in nursing studies, and SSM has a background in philosophy. Although both were familiar with the CEC service through their academic involvement, they were not members of the CEC team and did not hold any supervisory or evaluative role with respect to the participants. Each interview lasted approximately 30–45 min and included 18–20 questions covering key domains relevant to the evaluation of the CEC service (see Table S1). These domains included A) moral reflection [3,14,15], the emotional impact of ethically complex situations [13], cooperation (interprofessional cooperation, shared decision-making) [3,5,16], management of complex medico-legal issues/cases [3], doctor-patient communication, shared treatment planning [7], patient care [3,15,17], case management [3,16,17], and overall evaluation of the CEC service. Before addressing the predefined domains, each interview began with a question focused on the participant’s direct experience, inviting them to recount an episode related to their interaction with the CEC service. In addition, during analysis, particular attention was paid to differentiating experiential narratives from reflective or prospective statements, which are discussed accordingly in the Results and Discussion. We also sought to assess the service indirectly through the interviewees’ narratives, avoiding overt evaluative questions that might, in a semi-structured format, introduce inhibition-related bias. To minimize memory-related distortions, only individuals who had interacted with the service within the previous two years were included.

The interviews were conducted in a dedicated space within the FPG to ensure participants’ privacy and to encourage them to express their thoughts and emotions freely. All interviews were audio-recorded and subsequently transcribed.

**Figure 1:**
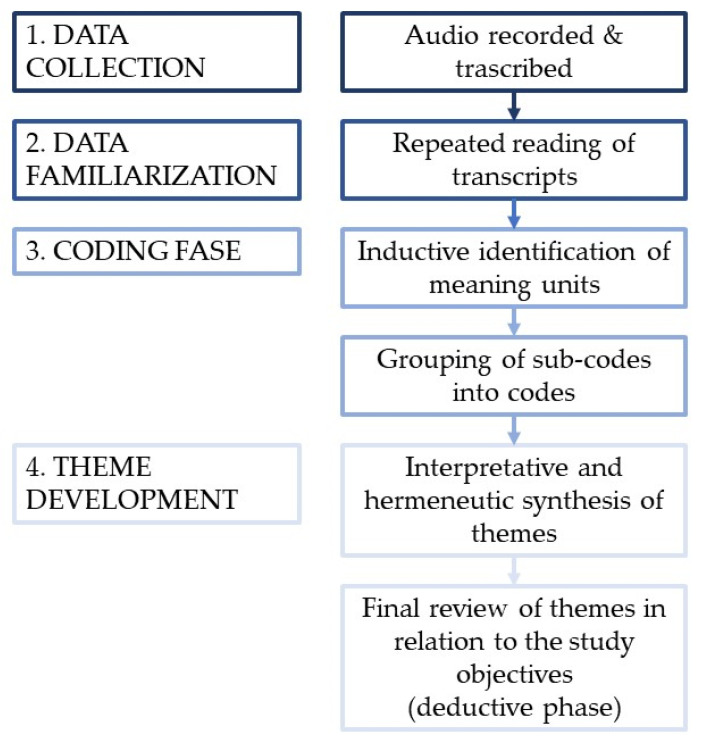
Methodological phases.

We then proceeded with a thematic analysis of the transcripts, beginning with the development of codes and sub-codes, through an inductive analysis (to capture and explain small units of meaning within the data), these were later grouped—through a deductive process—into broader themes relevant to the study’s objectives. The reference framework is defined by Braun et al. as Reflexive Thematic Analysis [18] and requires the researcher to take an active interpretative role in understanding and identifying the meanings that emerged from the narrative data. Figure 1 summarizes the main stages of the qualitative analytical process, from data collection and familiarization to inductive coding, analytical structuring, and interpretative theme development.

According to Braun et al. [18], coding represents the first real step of analysis. Codes are small semantic units generated through close reading and understanding of the interview transcripts, as well as through the grouping of similar concepts. For each code, its absolute frequency and its frequency relative to the number of interviews was calculated (see Table S2). Before proceeding to thematic analysis, codes were clustered into sub-codes, which in turn were combined to form primary codes. In qualitative analysis, the frequency of a code does not imply a hierarchy; rather, it is the interview process itself that determines which codes are relevant and how they contribute to the construction of themes. Furthermore, it is important to emphasize that many codes are inherent in the question itself, and therefore may show high absolute and relative frequencies because they are prompted by the interview structure itself.

In presenting the themes, although not commonly adopted in qualitative analysis, we reported the codes that most informed the development of each theme, along with their frequency, to enhance transparency in the theme construction process. Four themes were developed from the analysis of the codes. For each theme, the corresponding coded textual reference and their relative frequencies are reported; however, the organization of themes follows a hermeneutic and interpretative logic rather than a quantitative one.

The team’s contributions were as follows: Spagnolo A.G., Refolo P., and Masilla S.S. conceived and designed the study; Corsano B. identified, for each clinical area, the healthcare professionals to be involved and facilitated their recruitment; Todini C. and Masilla S.S. conducted the interviews; Masilla S.S. performed the initial textual analysis to identify the codes; Raimondi C., Refolo P., Todini C., and Masilla S.S. contributed to the thematic development of the results. Masilla S.S. drafted the first version of the manuscript, which was critically reviewed and approved by all authors.

The sample consists of healthcare professionals currently working at FPUG with patient care-related roles who have requested or been involved in a CEC at least once in the last two years (see Table 1).

Informed consent for study participation and data processing was obtained from all participants. The sampling frame was based on the clinical areas indicated in previous CEC service reports at FUPG, derived from retrospective analysis of internal databases [3,7,8]. These areas included surgery, internal medicine and infectious diseases, neurology, obstetrics and gynecology, pediatrics and neonatology, resuscitation and intensive care, and palliative care. Each area was intentionally represented to ensure diversity in the professional experience of the enrolled participants. The present report includes information on each participant’s profession, academic role, and annual frequency of involvement with the CEC service.

Since this was a predominantly exploratory study, no formal sample sizing was required; instead, a convenience sample was recruited until saturation was reached, resulting in a total of 28 participants. Case (or data) saturation refers to the point in qualitative research at which further data collection no longer yields new relevant information, themes, or conceptual categories in relation to the research questions. It indicates that the sample is adequate and that the collected data are sufficiently rich and repetitive to support robust analysis [19].

Seventy-five percent of the sample consisted of physicians, and 35% were other professionals, including nurses, health workers, psychologists, social workers, and chaplains/spiritual caregivers.

## 4. Results

### 4.1. Theme 1: Benefits of CEC for Clinical Practice

As stated by the ASBH, the ultimate purpose of CEC is to support the healthcare team in providing care to patients and their families [1]. Interviewees identified the elements through which the service positively impact clinical practice.

#### 4.1.1. Facilitating Interdisciplinary Processes

Several sub-codes converged to form what emerged as the most frequent code in the interviews (92): “facilitation of interdisciplinary processes”. This facilitation occurs through the promotion of interdisciplinary meetings involving the most appropriate professionals and through support for shared care planning. In this context, the consultant’s mediation role is widely recognized by participants (reported with a relative frequency of 64%), both because of the consultant’s overarching, global view and for the third-party role already discussed.

The Consultant is described as a “helmsman” in difficult decision-making processes:

“Clinical ethics consulting charts what can be a sort of shared course for the ship. In very complex cases, the consultant is the helmsman”

(Interview 20)

The mediation approach is closely linked to communication skills, reflected in the consultant’s ability to adopt a language that is appropriate for effective dialogue with each stakeholder.

Many participants state that the structured involvement of the team, with the presence of a clinical ethics consultant, promotes greater multidisciplinarity in the management of complex cases; the emergence of often marginalized perspectives (e.g., nursing concerns); the balancing of internal hierarchies and diverse specialist approaches.

#### 4.1.2. Prevention of Overtreatment

The prevention of unreasonable treatment obstinacy emerges from the combined analysis of two codes: “support in the assessment of treatment proportionality” (68%) and “end-of-life support” (21%). The topic is widely discussed in the literature [20], which is why the investigator also stimulated reflection on the issue. Interviewees described how ethics discussions helped them reflect on the appropriate level of intervention, particularly in situations characterized by poor prognosis or end-of-life decision-making. In these cases, the consultation was experienced as a space in which ongoing treatments could be reconsidered and adjusted in light of clinical goals and anticipated benefits.

“My expectation was to be able to have help in focusing on the right level of intervention, thus slightly fine-tuning the treatments, the diagnostic-therapeutic interventions that we obviously continued to implement despite knowing that the trend was negative”

(Interview 19)

Overall, in interviews where this topic emerged, the CEC service was described as supporting clinicians in recognizing and preventing overtreatment.

#### 4.1.3. Support in Shared Care Planning

A central element concerns shared care planning. This theme emerges frequently in the interviews (57%), participants associate Shared Care Planning with the operational tool known as the “Shared Document of Ethical-Care Orientation” [7]. This shared document is drafted by the consultant following focused multidisciplinary meetings with the care team and other relevant stakeholders and is subsequently shared with the patient or family members. In some cases, it has also been used to update Advance Treatment Directives previously filed by the patient.

Many participants emphasized the need for targeted support and dedicated expertise in shared care planning, a role that is often associated with the clinical ethics consultant.

The need for a shared decision-making process within the framework of shared treatment planning is considered important both from a medico-legal perspective (32%) and for supporting the management of complex cases (29%). In such situations, the relevant codes identify the caregivers’ need to navigate complex situations without fear of legal repercussions, allowing them to focus exclusively on the patient’s well-being.

#### 4.1.4. Facilitating Patient Engagement

Some of the outcomes of CEC are not the primary reason for requesting a consultation, but a significant collateral benefit is certainly the improvement of communication with patients, which in turn improves their adherence. The consultant’s role in this area has already been noted in the context of communication skills. From the participants’ perspective, this theme generated two highly frequent codes: “communication with the family” (68%) and “communication with the patient” (25%).

“I wanted help in making the family more aware of this situation and helping them accept the possibility that, faced with an acute event, they would have to decide whether to intensify treatment or withdraw”

(Interview 19)

Improving concordance therefore largely depends on a well-structured dialogue mediated by designated professionals such as the clinical ethics consultant. It is precisely in this regard that communication with family members appears to have greater impact on healthcare providers: it emerged in 68% of interviews, compared with 25% for communication with the patient. The consultant acts as a “translator” between technical language and personal understanding:

“The consultant has the ability to summarize and better understand medical jargon, also simplifying clinical and healthcare pathways”

(Interview 28)

#### 4.1.5. Improved Overall Patient Care

Many professionals recognize that ethics consulting improves comprehensive patient-centered care by aligning clinical decisions, personal values, family resources, and the care context. The sub-theme is supported by two key codes: “global, integrated vision” (36%) and “support in addressing the needs of the patient and family” (21%).

The presence of a consultant in care planning processes ensures that attention is also paid to the patient’s social/family, housing, and cultural context: these factors often introduce issues extending beyond the strictly clinical domain. Providing comprehensive and integrated care, therefore, requires moving beyond narrow specializations towards a broader, more comprehensive vision that encompasses all treatment scenarios.

#### 4.1.6. Indirect Emotional Support

Emotional support was a highly prevalent topic in interviews (71%). Although it is not usually the primary reason for requesting a clinical ethics consultation, many caregivers noted that it becomes one of the consultation’s significant effects through decision-making support it provides. In particular, the presence of an external eye and the opportunity to jointly plan the therapeutic process help relieve caregivers of the fear of legal reprisals or of failing to do the right thing for the patient. This, in turn, reduces the so-called “decisional isolation” [21].

Complex choices often manifest as a “moral distress” [22] especially among professionals who are less involved in formal decision-making processes but more closely involved in bedside care, such as nurses. The consultant’s involvement also helps resolve situations of inter-hierarchical conflict often associated with moral distress.

Interviewees emphasized that emotional support is typically delegated to specialists widely present within the hospital’s departments, and that it is not the primary goal of the CEC. For this reason, we decided to refer to it as “indirect emotional support.”

### 4.2. Theme 2: Proposals to Improve the Evaluation of the CEC Service

We explored possible strategies for evaluating the service, responding to gaps in the literature [23] and the lack of validated tools [24,25].

During the interviews, several practical proposals emerged, focusing on primarily on three areas: tools for collecting feedback, monitoring shared quantitative and qualitative indicators, audit, and debriefing processes.

The corresponding codes were grouped into three different strategies: quantitative (43%), qualitative (32%), and semi-qualitative (25%). Participants noted that their technical-scientific mindset tends to favor objective and quantifiable data. At the same time, some acknowledged that purely quantitative data leaves little room for reflection and narrative analysis: elements that are essential for capturing the complexity of a service like CEC.

#### 4.2.1. Tools for Collecting Feedback

Interviewees suggested that service evaluation should include periodic questionnaires addressed to the requesting healthcare professionals and, where possible, also to patients and family members.

“It would be useful to have a short form to fill out immediately after the consultation, so we can indicate whether it was clear and timely”

(Interview 05)

The purpose of this strategy is not only to gather short-term feedback, but also to establish a follow-up process for patients who remain under care at the hospital in order to assess the medium and long-term impact of the CEC service on the therapeutic pathway.

The items included in this feedback collection are those related to stakeholder satisfaction, adherence to the requester’s expectations, and overall satisfaction with the service.

Extending the evaluation process to include patients and their families would provide a more comprehensive understanding of the impact of the CEC service on clinical practice.

#### 4.2.2. Monitoring Quantitative and Qualitative Indicators

According to interviewees, service evaluation should be based on both quantitative indicators (e.g., number of requests, response times, number of requesting UOCs, analysis on service costs in relation to interventions assessing therapeutic proportionality of treatments) and qualitative indicators (e.g., level of team involvement, satisfaction, perception of support). Numerous interviewees highlighted the importance of “knowing how many cases were processed and how quickly interventions were carried out” (Interview 09) as well as the importance of “having a space to understand whether counseling helped them feel less alone in difficult decisions” (Interview 03).

#### 4.2.3. Audits, Debriefing

One of the subcodes related to improving the counseling service concerns audits, which appeared in 25% of interviews. Professionals proposed that evaluation should not be limited to one-off assessments (at the end of each consultation) but should also include periodic reviews, aimed at the continuous improvement the service. Proposed strategies include monthly/quarterly review meetings of the consulting service, analyzing the most complex cases, identifying recurring themes, “lessons learned,” and reviewing operating procedures. This aspect will be further addressed in the next section.

### 4.3. Theme 3: HCP Evaluation

The interviewees’ evaluation is expressed on three levels: Efficiency, Effectiveness, and Satisfaction.

Efficiency relates primarily to logistics. Participants discussed this theme through the codes: evaluation of the consultant’s competence (32%), timeliness (43%), and service coverage (21%). Where this theme emerged, participants generally recognized good specific expertise, including knowledge of clinical data and excellent clinical ethics evaluation tools. Nevertheless, 14% of participants suggested implementing consultant training, particularly regarding unit-specific clinical issues that vary across hospital areas. Regarding timeliness, 11 of the 14 interviewees who addressed this issue emphasized the need to better align consultation times with clinical workflow by improving the speed at which reports are delivered. Service coverage—understood as the presence of consultants in all areas of the hospital—was discussed in eight interviews: participants highlighted the need to expand the presence of consultants even in units with lower demand. Indeed, they noted that now there are some areas that are not covered by the CEC service at all.

Effectiveness refers to the service’s outcomes, particularly its impact on the care process. It is emerged in the interviews through several sub-codes related to its impact on care processes, which we have already discussed in the context of the consultant’s identity: indirect emotional support (71%), support in addressing the needs of patients and family members (21%), support in managing complex cases (29%), end-of-life support (21%), and facilitation of concordance (68% of family members, 25% of patients). Across all clinical areas represented in the study, respondents unanimously reported that the service has a consistently positive impact on care processes.

Satisfaction is an area of inquiry that we chose not to address directly, given the nature of the study (face-to-face interviews), as responses might have been affected by inhibition bias. However, we gathered all spontaneous comments made by the interviewees regarding the service’s ability to meet their expectations and their overall satisfaction with the CEC. Such comments were present in half of the interviews and were grouped under the code “satisfaction” (50%). In all cases, participants reported being fully satisfied with the CEC outcomes.

### 4.4. Theme 4: CEC Service Improvement Strategies

From the qualitative analysis of the interviews and the emerged codes, strategies for improving the CEC service cluster around two key dimensions: organizational aspects and training needs. The most frequently cited factors are as follows: increasing the presence of consultants on the wards (29%), increasing the number of consultants (36%), organizing audits or debriefings (25%), training of healthcare personnel (39%), and training of consultants (14%).

Given the short reporting deadlines and the wide distribution of the service across numerous hospital units, interviewees recommend increasing the number of dedicated consultants. The suggested increase should be not only numerical but also qualitative: several participants emphasized the need for dedicated consultants whose primary role is ethics consultation, allowing them to be consistently available to teams rather than responding solely to individual requests. According to some interviewees (8), this continuous presence could be granted through regular department meetings (audits), or through debriefings conducted after case resolution.

“After six months, a meeting with the entire healthcare team would be useful to discuss the most sensitive cases and see if anything can be changed”

(Interview 12)

Many professionals report that ethical reflection is not yet an integral part of clinical training, and they see the consultant as compensating for this gap. Fifteen interviews explicitly refer to the consultant as playing a role in clinical ethics training. Implementing training sessions is viewed as an excellent strategy for raising healthcare personnel’s awareness of the use of the CEC service and for stimulating demand in areas of the hospital that are currently less sensitive to the such approaches. In addition to targeted training, information and awareness campaigns within the hospital are recommended to foster an interdisciplinary approach and a care culture attentive to values.

Eight interviewees highlighted the need to further training for consultants themselves, so they can better address the specific needs of the hospital’s various specialties. In this regard, several professionals emphasized the importance of consultants developing specialized expertise in specific clinical areas, making the consultation approach more aligned to the needs of the requesting team.

Staff members recognize the service as an important tool for decision-making support and mediation, but they also emphasize the need to consolidate its operational efficiency, increase its institutional visibility, and further promote an ethical culture within the hospital.

### 4.5. Additional Findings

The semi-structured interviews also yielded findings that extend beyond the original scope of the research. In this section, we focus specifically on findings related to the role of the consultant, as they emerged from discussions with healthcare professionals/as they emerged from the interview.

The clinical ethics consultant emerges as a complex and multifaceted professional, positioned at the intersection of ethical reflection, clinical practice, and relational dynamics. This role extends far beyond a purely technical or bureaucratic function and requires epistemic, communicative, and mediation skills, as well as the ability to continuously balance neutrality with active engagement in decision-making processes.

One particularly informative code—“global vision”—appeared 12 times in the interviews (relative frequency: 36%) without being prompted by the interview guide. This code highlights the consultant’s capacity to harmonize diverse perspectives and underscores the integrative vision that consultants bring to the multidisciplinary team, reinforcing their role as mediators.

#### 4.5.1. Training and Professional Background

The background of the clinical ethics consultant was widely discussed in the interviews (mentioned 26 times) and reflected in the codes “Professional profile” (18%), “Basic training” (32%), and “Skills” (36%). Many interviewees emphasized the importance of specific training in bioethics and clinical ethics, supported by solid clinical competence and communication skills.

The dual ethical-clinical dimension was viewed as essential for understanding the concrete contexts in which decisions are made and for communicating effectively with clinicians. In several cases, interviewees also highlighted the importance of specialty-specific clinical expertise related to the requesting unit.

Five participants expressed explicit views regarding the consultant’s professional profile. Three stated that the consultation should be conducted by a physician, arguing that other background would represent a loss for the service. The remaining two were open to different profiles, provided that the consultant possesses adequate clinical expertise. Eleven interviews focused on basic training, almost unanimously expressing the need for an educational pathway grounded in healthcare practice and complemented by ethics training. Three interviewees were open to the involvement of philosophers as consultants, citing their analytical mindset as a valuable resource for decision-making processes.

Across interviews, communication and mediation emerged as the most frequently discussed competencies. Interviewees emphasized the importance of a professional capable of facilitating decision-making processes and mediating both intra-team conflicts and tensions between healthcare teams, patients, and families.

#### 4.5.2. Third-Party and Neutrality

A frequently discussed aspect was the consultant’s third-party status. The code of “third-party” appeared 11 times in the interviews, with a relative frequency of 36%. The consultant’s “external” position in relation to the team is perceived as a defining element of their professional identity.

This third-party status is not understood in a passive distance, but rather as the ability to offer a detached perspective (“an external eye”) that helps to illuminate elements not perceived by clinicians, who are often immersed in clinical and relational complexities. In conflict situations, this external position is a resource for balance and authority.

“I think a third-party figure is appropriate first and foremost because it offers another perspective. When you are immersed in the problem, you can’t see many aspects objectively”

(Interview 20)

The consultant’s third-party status is associated with the expectation of objectivity, which participants perceive as a guarantee fundamental for ensuring a sound decision-making process. Almost all participants (25/28) described the burden of personal and emotional involvement when making decisions with significant implications on multiple levels, often influenced by their role. In eleven cases, the consultant was seen as providing support in managing this involvement.

Neutrality also emerged as a key theme alongside third-party involvement, especially in the context of interdisciplinary processes. The literature describes this function as “moral space facilitation”: the consultant creates a protected and reflective environment that enables the different parties to reframe the problem and negotiate decisions collaboratively [2].

#### 4.5.3. Mediation and Facilitation

The clinical ethics consultant was frequently described as a mediator between divergent perspectives, whether between clinical specialties, across internal hierarchical levels, or between healthcare teams and families. The corresponding code appeared prominently in the interviews, with an absolute frequency of 28 and a relative frequency of 64%.

This mediation function was also evident in shared care planning and discussions with patients and family members. Interviewees described the consultant as helping to establish a common language, translating specialized concepts and facilitating mutual understanding. Themes such as shared care planning and communication with family members and patients intersect across multiple levels: they reflect the consultant’s role in interdisciplinary dialogue, relational mediation and the development of therapeutic alliances.

#### 4.5.4. Proportionality

A further central aspect of professional identity concerns their analytical and evaluative expertise in the area of therapeutic proportionality. Consultations are often requested to support the examination of whether treatments are proportional, that is, to help clarify/identify expected benefits, potential burdens, and patient values.

“I have requested CEC many times, for example, to discuss the usefulness or otherwise of performing PEG. I wanted to understand what was right, even morally, for the patient, what was too much or too little”

(Interview 11)

The code “support in the analysis of the proportionality of treatments” appears 29 times in the interviews, with a relative frequency of 68%. Less frequent are codes related to ethical or moral reflection (11) and “support in medical-legal issues” (9). Nevertheless, ethical expertise is perceived as a key component of the consultant’s contribution, providing a structured framework for discussing moral dilemmas, helping to distinguish between objective and subjective elements.

This role was considered particularly central in end-of-life contexts and shared care planning, where therapeutic proportionality is essential to avoid both unreasonable treatment obstinacy and therapeutic abandonment.

## 5. Discussion

From the analysis of the interviews, the service emerges as a tool that concretely impacts the quality of care, not only by promoting shared treatment planning and preventing unreasonable therapeutic obstinacy as Schneiderman states [20], but also by improving communication between healthcare providers, patients, and families in line with the experience reported by Spagnolo et al. [3]. Ethics consultation also proves to be an emotional support tool for caregivers, allowing them to share the burden of decision-making and reduce moral isolation that often accompanies more complex clinical decisions. In this sense, according to interviewers, the service would contribute to the prevention of moral distress, especially among those most exposed to the relational dimension of care, such as nurses as emerges from the work of McAndrew et al. [22]. Overall, the effect is greater team cohesion, improved interdisciplinary integration, and strengthened trust in the decision-making process [7]. At this level, the service is perceived as supporting patient Engagement, but even more so the family context, where caregivers feel the greatest need for support. Many of the challenges described by physicians were linked to the social and family dynamics, underscoring the need for further support in managing communication with families.

Regarding service evaluation strategies, interviewees express strong support for the introduction of structured monitoring and continuous feedback tools as emerges from the work of Haltaufderheide et al. [25]. These tools should be based on both quantitative indicators (response times, number of consultations, distribution by operational unit) and qualitative indicators (level of satisfaction, perceived impact, training value) [11]. Proposals for periodic audits and interprofessional debriefings also emerged as opportunities for collective learning and continuous improvement. At the same time, interviewees emphasized the importance of expanding service coverage by increasing the presence of consultants in hospital units and enlarging the team of dedicated professionals.

It also became evident that, although quantitative evaluation can help to objectify outcome analysis [12], they fail to fully capture the full complexity and multidimensional nature of the service, which operates simultaneously on ethic, clinical, and relational [17]. This very complexity calls for diverse and complementary evaluation approaches. In this study service evaluation—through the analysis of interviews—was examined using three indicators: efficiency, effectiveness, and satisfaction [26].

Efficiency concerns organizational aspects and encompasses the assessment of consultants’ competence, the timeliness of responses and reporting, and the coverage of the service. Participants generally acknowledged the consultant’s high level of competence and the appropriate use of ethics assessment tools; however, 14% suggested strengthening training to better respond to the specificities of different departments. Regarding timeliness, most interviewees noted the need to align response times with clinical workflow. A recurrent request also emerged for broader service coverage, areas as some hospital areas are still not adequately reached by the CEC service.

Efficiency is related to how well the service outcomes align with the needs of the clinical setting. However, evaluating it remains a complex task, as outcomes may not always correspond to the healthcare professionals’ expectations. For instance, communication processes can affect the amount of time required to build a relationship with the patient. How should such an outcome be interpreted? Would resolving a complex situation in less time than the department’s average performance be considered a positive or a negative result? Despite these challenges, assessing efficiency remains essential for understanding how well the service is integrated into hospital dynamics.

Effectiveness was assessed in terms of the service’s impact on care processes. Interviewees expressed unanimously positive opinions, citing indirect emotional support, assistance in managing the needs of patients and families, management of complex cases, support during the end-of-life phase, and facilitation of patient and family members’ Concordance with clinical decisions.

This aspect of service evaluation still requires further development and offers opportunities for future research. To measure the service’s effectiveness in generating tangible benefits for both the clinical team and patients, it will be essential to identify specific outcomes. This also emerges from the analysis of Crico et al. [27] about the evaluation of effectiveness of clinical ethics committees. These should assess not only user satisfaction (as discussed in the next section) but also the extent to which the service meets the particular objectives for which each consultation is requested.

Satisfaction, measured indirectly to minimize bias, was uniformly positive: half of the interviewees spontaneously provided comments indicating the service was closely aligned with expectations. Based on our analysis, however, staff satisfaction should not be considered the primary evaluation criterion, especially in cases of conflict between the consultant and the team or between the team and the patient. Rather, assessing participant satisfaction instead can help capture their experiences and understand their perspectives [26].

Overall, the service was perceived by healthcare professionals as being of high quality. This assessment is supported by consistently positive evaluations across the dimensions of efficiency, effectiveness, and satisfaction. Participants emphasized the consultants’ competence, the usefulness of ethics assessment tools, and the service’s positive impact on care processes, particularly in complex and end-of-life situations. Although some critical issues were identified—such as the need for improved training, better alignment with clinical timelines, and broader service coverage—these were generally framed as areas for enhancement rather than as fundamental shortcomings. Taken together, the findings indicate that, from the operators’ perspective, the service effectively meets clinical needs while leaving room for further improvement.

Looking ahead, the most widely shared improvement strategy is to invest in the clinical ethics training of healthcare personnel, so that the culture of ethical and clinical reflection becomes an integral part of the care pathway and not an extraordinary external intervention. At the same time, there is a recognized need for consultants to further specialize, developing specific skills tailored to specific clinical areas in order to make the consultation service increasingly targeted and operational. This is also understood from the report by Corsano et al. of the CEC service carried out in the NICU [8].

Although it is not within the objectives of the study, we considered it appropriate to present the data that emerged from the interviews regarding the role of the clinical ethics consultant.

The clinical ethics consultant emerges as a complex professional figure positioned at the intersection of technical knowledge and moral reflection, bridging the scientific and value-based dimensions [2]. Professionals perceive the consultant as a facilitator of decision-making processes and a mediator capable of creating a space for shared reflection where the team can constructively discuss moral dilemmas and difficult therapeutic choices. This emerges also from the analysis of Shelton, W. et al. [28]. Communication skills and the ability to maintain an active third-party position—that is, a neutral yet fully engaged—are recognized as distinctive traits of their role [29]. In this perspective, the notion of the consultant as an “active third party” does not imply a position of detachment or neutrality in a passive sense, but rather a form of engaged impartiality. Unlike purely facilitative roles, which focus primarily on structuring dialogue, the active third-party position combines mediation and ethical engagement, allowing the consultant to guide the process while remaining external to the clinical hierarchy. This stance enables the consultant to balance differing viewpoints, reframe conflicts, and support deliberation without assuming decision-making authority. As such, mediation and facilitation are not ends in themselves but instruments through which the consultant actively sustains a reflective moral space.

The findings also highlight a cultural limitation: the consultant’s ethical-philosophical dimension is often perceived only implicitly or regarded as secondary to their operational and facilitative role. Healthcare professionals tend to view the consultant primarily as a facilitator in decision-making processes or information sharing, rather than as a resource for ethical analysis of cases. Nevertheless, ethical expertise is perceived as a key component of the consultant’s contribution in supporting decision-making processes. The consultant provides a structured framework for discussing moral dilemmas. As a consequence, CEC is more often requested to address the management of complex clinical situations than to explore the ethical dilemmas that arise in clinical practice and in the practitioner’s own moral reflection [21]. This aspect suggests the need to strengthen training for healthcare professionals in bioethics to fully benefit from the analytical and reflective component of the consultation.

## 6. Limitations of the Study

Since the interview was conducted face-to-face, the presence of an interviewer may have inhibited participants and limited the spontaneity or completeness of their responses. To mitigate this limitation, interviews were conducted by sub-investigators who were involved in the service only as external observer.

One limitation of the study concerns the predominance of physicians among the participants, which may have influenced the findings by emphasizing perspectives specific to their professional role. Nevertheless, physicians represent the largest group of healthcare professionals at our hospital and are therefore key stakeholders in the use and evaluation of the service.

Another limitation concerns the temporal distance between the interviewee and the CEC experience: memory lapses or the reworking of past experience, too distant in time, may have affected how participants narrated the experience and perceptions.

Further limitations are linked to the subjective nature of data interpretation. The coding method is inevitably associated with the sensitivity and background of the researchers, even though it remains the most appropriate method for capturing participants’ perceptions within a qualitative study.

## 7. Conclusions

This qualitative study highlights the value of CEC at FPG as both an integrative component of care and a reflective space supporting healthcare professionals. The service helps guide clinical decisions toward greater therapeutic proportionality and a more authentic patient-centered approach, fostering balance among the technical, ethical, and relational dimensions of care.

The consultant emerges as a bridge between scientific knowledge and moral reflection, serving as a facilitator of interdisciplinary dialogue within the healthcare team and communication with patients and families. Their “active third-party” role fosters trust and team cohesion, mitigating decision-making isolation and moral distress. However, the consultant’s ethical and philosophical function remains only partially recognized, often overshadowed by the mediating role. This finding underscores the need for more comprehensive ethics education among healthcare professionals.

The analysis of the three evaluation indicators—efficiency, effectiveness, and satisfaction—confirms the overall positive impact of the service, while pointing to critical areas such as timeliness and coverage. The proposed improvements emphasize expanding service reach, strengthening dedicated staff, and implementing structured qualitative and quantitative monitoring tools.

Looking forward, the CEC stands out not only as a clinical support but also as an educational resource as it fosters a better ethical and professional culture of person-centered care of the institution. The qualitative findings reveal that the commitment of the staff involved in the counseling service has contributed to its growth over the years, while also fostering greater awareness among healthcare professionals of a patient-centered ethical clinical approach. The service’s activities align with the broader goal of promoting the humanization of healthcare settings and care processes.

## Figures and Tables

**Table 1:** Sample analysis.

SEX	#	%
Male	11	39
Female	17	61
**AGE**		
35–44	1	4
45–54	16	57
55–64	11	39
**PROFESSION**		
physician	21	75
Nurse	3	11
other professions	4	14
**ACADEMIC ROLE**		
Professor	17	61
Researcher	2	7
None	6	32
**EXPERIENCE WITH THE CEC SERVICE ^2^**		
rare < 1 per year	1	4
occasional 2–5 per year	8	29
frequent > 5 per year	11	39
regular ^1^	8	29

^1^ when the CEC is included in the Hospital clinical procedures. ^2^ the number of times the subject participated in a CEC.

## Data Availability

The original contributions presented in this study are included in the article/Supplementary Material. Further inquiries can be directed to the corresponding author.

## Supplementary Materials

The following supporting information can be downloaded at: https://www.mdpi.com/article/10.3390/healthcare14030395/s1, Table S1: semi-structured interview outline; Table S2: Frequency of code.

## Abbreviations

The following abbreviations are used in this manuscript:

CEC	Clinical Ethics Consultation
FPG	Fondazione Policlinico “A. Gemelli”
ASBH	American Society for Bioethics and Humanities
