# Supplementary material for: The VALUE Study: Exploring the Value of a Clinical Ethics Consultation Service at the “A. Gemelli” Hospital
Source: Healthcare (Basel). 2026 Feb 4;14(3):395. doi: 10.3390/healthcare14030395 (PMC12897271; doi:10.3390/healthcare14030395)
Supplement: Supplementary file 1 [file healthcare-14-00395-s001.zip › healthcare-4063763-supplementary.pdf]

The VALUE Study: Exploring the Value of a Clinical Ethics Consultation Service

Supplementary

Table S1: semi-structured interview outline

Macro-tema	Domanda	Rilancio
**Introduzione**	1) (per un soggetto partecipante) come sei stato coinvolto nella CEC? Cosa sapevi in merito a questo servizio?<br><br>(Per un soggetto richiedente) cosa ti ha spinto in passato a richiedere una CEC? Cosa ti aspettavi dal consulente?	
	2) Quali sono gli aspetti più complessi dell'atto di cura?	a) Ti capita di percepire il peso delle sue azioni/decisioni in ambito lavorativo?
**Cura del paziente**	3) Per quali aspetti (se vi sono) nella cura del paziente ritieni essere utile la CEC?	b) come valuti l'impatto della CEC nella gestione di casi complessi?<br>c) Ti è mai capitato di chiedere aiuto ad un consulente per un problema medico/legale o per un dubbio di natura deontologica?
	4) come valuti l'apporto del consulente nella riflessione circa la proporzionalità terapeutica di un trattamento?	
	5) Qual è l'impatto del consulente nella pianificazione condivisa delle cure?	
**Comunicazione**	6) In merito alla pianificazione condivisa delle cure, un ruolo cardine assume la comunicazione m/p. Come definiresti l'impatto del consulente in questo ambito?	d) Ritieni che la presenza del consulente possa facilitare il processo di comunicazione m/p?
	7) In che modo impatta la presenza di un consulente nell'approccio con i familiari?	e) Ti sei mai sentito supportato dal consulente nel dirimere situazioni complicate dalle richieste dei familiari?
	8) Quanto è importante la presenza di un consulente nel confronto tra colleghi?	f) Quale ruolo assume il consulente nel dialogo tra le diverse gerarchie di potere?
**Cooperazione**		
	9) Quale apporto fornisce il consulente all'interno dell'equipe multidisciplinare?	g) Quale ruolo può assumere il consulente nel dialogo interprofessionale?
	10) Dopo la fase del confronto, i membri dell'equipe multidisciplinare secondo le proprie competenze operano per la realizzazione del miglior bene per il paziente. Qual è l'apporto del consulente (laddove vi fosse) in questa cooperazione?	h) Trovi utile lo strumento del documento condiviso di orientamento etico-clinico assistenziale ai fini della cooperazione per la gestione di un caso complesso?

	11) Qual è l'impatto del consulente nella cooperazione tra le diverse gerarchie intraospedaliere?	i) Trovi utile la presenza di un consulente dinanzi ad incomprensioni che possono manifestarsi tra la struttura (o la direzione sanitaria) e i curanti?
**Riflessione morale**	12) Quanto può impattare sul piano emotivo un quesito etico/morale complesso per l'operatore?	
	13) In che modo il consulente impatta sulla riflessione morale che accompagna l'atto di cura?	j) Hai mai sentito il bisogno di un supporto nel dirimere un problema etico? Perché?
	14) Ritieni che l'expertise offerta dalla CEC sia in grado di supportare i curanti nella riflessione morale?	I) Riconosci nel consulente specifiche competenze di supporto per la riflessione morale?<br>m) Ritieni che la CEC possa avere un ruolo nella prevenzione del moral distress?
**Valutazione del servizio CEC**	15) Sapresti indicarmi punti di forza o debolezza del servizio di CEC?	n) Cosa dovrebbero migliorare o custodire in futuro i consulenti?
	16) Quali strategie potrebbero essere adottate al fine di valutare un servizio così complesso?	o) Ritieni più appropriato un approccio qualitativo o quantitativo nei sistemi di valutazione del servizio CEC?
**Conclusione**	17) Aggiungeresti altro ai temi trattati nella nostra discussione?	

Table S2: Frequency of code

Codice	Sottocodice	Frequenza assoluta	Frequenza relativa
**Facilitazione del processo decisionale**	Supporto nell'analisi sulla proporzionalità dei trattamenti	29	68%
	Supporto nella riflessione etica	11	36%
	Supporto dinanzi a problematiche medico/legali	9	32%
**Facilitazione dei processi interdisciplinari**	Coinvolgimento dell'equipe	15	46%
	Pianificazione condivisa delle cure	26	57%
	Mediazione	28	64%
	Visione globale/integrata	12	36%
	Terzietà/neutralità	11	36%
**Impatto sui processi di cura**	supporto emotivo indiretto	22	71%
	Supporto nella presa in carico dei bisogni del paziente e dei familiari	6	21%
	Supporto nella gestione di casi complessi	11	29%

	Accompagnamento nel fine vita	6	21%
	facilitare la compliance: comunicazione con la famiglia	26	68%
	Facilitare la compliance: comunicazione con il paziente	8	25%
**Background**	Profilo professionale	5	18%
	Formazione di base	11	32%
	Competenze	10	36%
**Valutazione del servizio**	Tempestività	14	43%
	Capillarità	8	21%
	Consapevolezza dell'operatore	5	18%
	Partecipazione dell'operatore	4	14%
	Valutazione della competenza del CEC	10	32%
	Soddisfazione dell'operatore	21	50%
**Strategie di valutazione**	valutazione quantitativa	13	43%
	valutazione qualitativa	11	32%
	Valutazione semi/qualitativa	7	25%
**Strategie di miglioramento del servizio**	Formazione del personale	15	39%
	Aumentare la presenza del consulente nei reparti	11	29%
	Aumentare il numero dei consulenti	10	36%
	Audit/debriefing	8	25%
	Implementare la formazione del consulente	4	14%
